# Automated classification of elongated styloid processes using deep learning models-an artificial intelligence diagnostics

**DOI:** 10.3389/froh.2024.1424840

**Published:** 2025-01-20

**Authors:** Anuradha Ganesan, N. Gautham Kumar, Prabhu Manickam Natarajan, Jeevitha Gauthaman

**Affiliations:** ^1^ Department of Oral Medicine & Radiology, SRM Dental College, Bharathi Salai, Chennai, India; ^2^ Department of Periodontics, Madha Dental College & Hospital, Chennai, India; ^3^ Department of Clinical Sciences, Center of Medical and Bio-allied Health Sciences and Research, College of Dentistry, Ajman University, Ajman, United Arab Emirates

**Keywords:** deep learning, panoramic radiography, convolutional neural networks, styloid process, diagnosis

## Abstract

**Background:**

The styloid process (SP), a bony projection from the temporal bone which can become elongated, resulting in cervical pain, throat discomfort, and headaches. Associated with Eagle syndrome, this elongation can compress nearby nerves and blood vessels, leading to potentially severe complications. Traditional imaging-based methods for classifying various types of elongated styloid processes (ESP) are challenging due to variations in image quality, patient positioning, and anatomical differences, which limit diagnostic accuracy. Recent advancements in artificial intelligence, particularly deep learning, provide more efficient classification of elongated styloid processes.

**Objective:**

This study aims to develop an automated classification system for elongated styloid processes using deep learning models and to evaluate the performance of two distinct architectures, EfficientNetB5 and InceptionV3, in classifying elongated styloid processes.

**Methods:**

This retrospective analysis classified elongated styloid processes using Ortho Pantomograms (OPG) sourced from our oral radiology archives. Styloid process lengths were measured using ImageJ software. A dataset of 330 elongated and 120 normal styloid images was curated for deep learning model training and testing. Pre-processing included median filtering and resizing, with data augmentation applied to improve generalization. EfficientNetB5 and InceptionV3 models, utilized as feature extractors, captured unique styloid characteristics. Model performance was evaluated based on accuracy, precision, recall, and F1-score, with a comparative analysis conducted to identify the most effective model and support advancements in patient care.

**Results:**

The EfficientNetB5 model achieved an accuracy of 97.49%, a precision of 98.00%, a recall of 97.00%, and an F1-score of 97.00%, demonstrating strong overall performance. Additionally, the model achieved an AUC of 0.9825. By comparison, the InceptionV3 model achieved an accuracy of 84.11%, a precision of 85.00%, a recall of 84.00%, and an F1-score of 84.00%, with an AUC of 0.8943. This comparison indicates that EfficientNetB5 outperformed InceptionV3 across all key metrics.

**Conclusion:**

In conclusion, our study presents a deep learning-based approach utilizing EfficientNetB5 and InceptionV3 to accurately categorize elongated styloid processes into distinct types based on their morphological characteristics from digital panoramic radiographs. Our results indicate that these models, particularly EfficientNetB5, can enhance diagnostic accuracy and streamline clinical workflows, contributing to improved patient care.

## 1 Introduction

The styloid process (SP) is a cylindrical bony projection from the inferior part of the petrous temporal bone and is positioned laterally in the neck in between the carotid arteries and the internal jugular vein and it plays a crucial role in the oropharyngeal complex's movement. Embryonically, it originates from Reichert's cartilage with ossification starting from the third trimester of pregnancy and continues through the first ten years of life. Various muscles and ligaments originate from the styloid process, contributing significantly to oropharyngeal movements. Elongation of styloid process is seen in various studies and prevalence ranges between 3.3% to 84.4% (1).

The first case of elongated styloid process (ESP) was described by Marchetti in 1,652 and later, Eagle, an otorhinolaryngologist described a syndrome characterized by occurrence of ESP and orofacial pain. It is characterized by episodes of pharyngeal pain referred to different areas of cervicofacial area (2).

Symptoms associated with elongation of the styloid process are diverse, encompassing cervical pain, throat discomfort, earaches, sensation of a foreign object in the throat, pain triggered by changes in head position, headaches, discomfort in the cervicofacial region, pain during swallowing, shoulder discomfort, and a feeling of throat obstruction. The elongated styloid process (ESP) may induce these symptoms through several mechanisms, including compression of the glossopharyngeal nerve, vagus nerve, or branches of the trigeminal nerve, pressure on the carotid vessels leading to carotidynia or carotid artery syndrome, degenerative or inflammatory changes in the tendinous portion of the stylohyoid ligament insertion known as insertion tendinitis or hyoid syndrome, or rheumatic styloiditis resulting from pharyngeal infection. Severe complications like transient ischemic attack or stroke can also occur due to compression of carotid artery (3). The normal length of SP is approximately 20–30 mm and the length of SP is measured as the distance from the point where styloid process leaves the tympanic plate to the tip of the process. Styloid processes measuring more than 30 mm are considered as elongated and can be seen unilaterally or bilaterally. An elongated styloid process is considered as an incidental finding that is encountered during routine dental checkups and radiographic investigations. The ligaments ossified are also measured along with styloid process as part of SP. The precise cause of elongation of the styloid process remains uncertain (4). Painful symptoms may be attributed to prior trauma resulting in a fracture of the styloid process or from previous tonsillectomy. Other proposed theories include congenital elongation, partial or complete ossification of the stylohyoid ligament, elongation at the cartilaginous junction of the tympanohyale and stylohyoid due to delayed ossification, post-trauma changes leading to reactive hyperplasia, and a potential association with early onset of menopause (5).

Radiologically the elongated styloid processes have to be assessed in detail which will enable us to differentiate it from neurological conditions like glossopharyngeal neuralgia, recurrent headaches, neck pain, migraine and dizziness. Various authors employ diverse radiographic approaches to study the elongated styloid process (ESP), such as lateral head and neck, posteroanterior skull, exaggerated Towne's, orthopantomography (OPG), lateral oblique of the mandible, anteroposterior skull, Cone Beam computed tomography and computed tomography. Computed tomography scans can define the length, angulation, and anatomic relationship of the styloid process. Nevertheless, orthopantomography is commonly favoured. OPG is a two-dimensional imaging technique which is considered as the first line of radiographic diagnosis for extraoral dento maxillofacial complex. Due to the ease of availability, lower radiation dose and cost-effectiveness when compared to a CT, OPGs are commonly preferred for evaluating anomalies of the orofacial region. It can be performed and interpreted with ease. The elongation of SP can also be identified in an OPG very effectively (6–9). Accurate classification of elongated styloid processes into different types based on their morphology is essential for appropriate clinical management, including diagnosis and treatment planning. Langlais et al. proposed the classification of ESP according to morphology as follows: Type I: uninterrupted; Type II: pseudo-articulated; and Type III: segmented (10). Traditionally, the classification of elongated styloid processes has relied on manual examination of digital panoramic radiographs by skilled radiologists or clinicians. However, this process is labour-intensive, time-consuming, and prone to subjective interpretation, leading to potential inconsistencies and inaccuracies in diagnosis. Moreover, traditional classification methods may overlook subtle variations in elongated styloid processes, which can impact treatment decisions and patient outcomes.

Artificial intelligence (AI) is revolutionizing dental radiology and primarily utilized for image analysis and interpretation, treatment planning, patient education, and research. AI algorithms are adept at analyzing dental radiographs to detect abnormalities and through deep learning techniques, these algorithms recognize patterns and features in images, providing dentists with more accurate and timely diagnoses. This not only improves patient care but also optimizes treatment outcomes. Quality assurance is crucial in dental radiology, and AI helps ensure the accuracy and reliability of radiographic images. By identifying errors such as artifacts and exposure issues, AI algorithms improve the overall quality of radiographic examinations, enhancing diagnostic confidence (11). A number of studies have successfully employed AI algorithms for measuring morphometric indices of the skull, including landmarks of the maxilla and mandible. They provide accurate data with high reliability and precision which can be used to differentiate normal from abnormal findings. In recent years, there has been growing interest in leveraging advanced computational techniques, particularly deep learning to automate the classification of medical imaging data. Deep learning models, such as convolutional neural networks (CNNs), have shown remarkable capabilities in extracting meaningful features from images and accurately classifying complex patterns (12–14). Image classification and feature extraction are performed with high precision that transform input images into useful output data which can be received in the form “image categories”, “object locations”, and “pixel labels”. These deep learning classifiers use numerous convolutional image layers followed by fully connected layers which can detect objects with accuracy comparable to or better than human performance. By harnessing the power of deep learning, there is potential to develop more efficient and reliable systems for classifying elongated styloid processes. The present study was aimed to use Deep Learning Models and to evaluate the performance of two distinct deep learning architectures, namely EfficientNetB5 and InceptionV3, in classifying and diagnosing elongated styloid processes.

## 2 Materials and method

The traditional methods of identifying the elongated styloid process and classifying them has been difficult as it has to accommodate the inherent variability in digital panoramic radiographs due to factors such as varying image quality, patient positioning, and anatomical differences among individuals. This limited adaptability can hinder their performance in accurately classifying elongated styloid processes across diverse patient populations. Also it might be difficult to capture the intricate relationships present in the images of elongated styloid processes. As a result, it might not be possible to discern subtle patterns or variations crucial for accurate classification, leading to suboptimal performance.

In the present study the proposed methodology for classifying different types of elongated styloid processes begins with the collection of real-time digital panoramic radiographs from clinical sources (archives from our oral radiology department) These images serve as the basis for training and testing our deep learning models. The present study was formulated according to the ethical standards of the Helsinki Declaration and approved by the Institutional review board. All methods were performed in accordance with the relevant guidelines and regulations as per ethics approval. Due to the retrospective nature of the study, the need for informed consent was waived by the Institutional Review Board Committee of SRM Dental college.

A total of 931 orthopantomograms (OPG) were included in the study. All the radiographs were taken from male and female patients who visited the dental O. P for routine dental checkup and examination. Digital radiographs of good resolution were included. However, images of patients below 18 years, duplicate images, images with distortion and artifacts were excluded. Images which have overlapping of other structures and thus having minimum visibility of styloid processes were excluded. Images with poor positioning, low resolution and images of patients with surgical defects or trauma in the maxilla and mandible were excluded from the data set. Thus, the total number of 938 was regressed to 330 OPG images. A single calibrated observer selected orthopantomograms (OPGs) for assessing the styloid process, following the specified inclusion and exclusion criteria. Using ImageJ software (National Institute of Health, Maryland, USA), the observer measured the length of the styloid process as a linear distance between the point where it extends from the temporal bone to its tip. To reduce potential bias or errors from examiner fatigue, only 20 radiographs were assessed per day.

Subsequently, we curated a dataset consisting of 330 images both unilaterally and bilaterally showcasing elongated styloid processes and 120 images displaying normal styloid processes. This dataset was divided into training and test sets as follows: a total of 20% of the radiographs (90 images) were set aside as a “test set”. This ensured that the testing data set only contained images of novel radiographs that have not been encountered by the model during training. The remaining 360 radiographs were used for the training and validation set. The SP which measured 20–30 mm from the point where styloid process leaves the tympanic plate to the tip of the process were considered as “Normal” and those which measured more than 30 mm were considered as “Abnormal”. Further, the curated dataset of Images was labelled according to the classification by Langlais et al. as: “Normal”, “Pseudo Articulated”, “Segmented”, or “Uninterrupted” and were further segregated between bilateral elongated styloid processes, left side alone, and right side alone. Type I SP had an uninterrupted morphology along the course of its entire length, type II SP was connected to the hyoid bone to form a “pseudo articulation” and type III SP contained a number of segmentations along its course with an overall length of more than 30 mm. Prior to model training, a crucial preprocessing step was employed to enhance the quality of the images and remove any irrelevant details. This involved applying a median filter for noise removal and resizing the images to a standardized dimension, ensuring consistency across the dataset.

**Figure 1:**
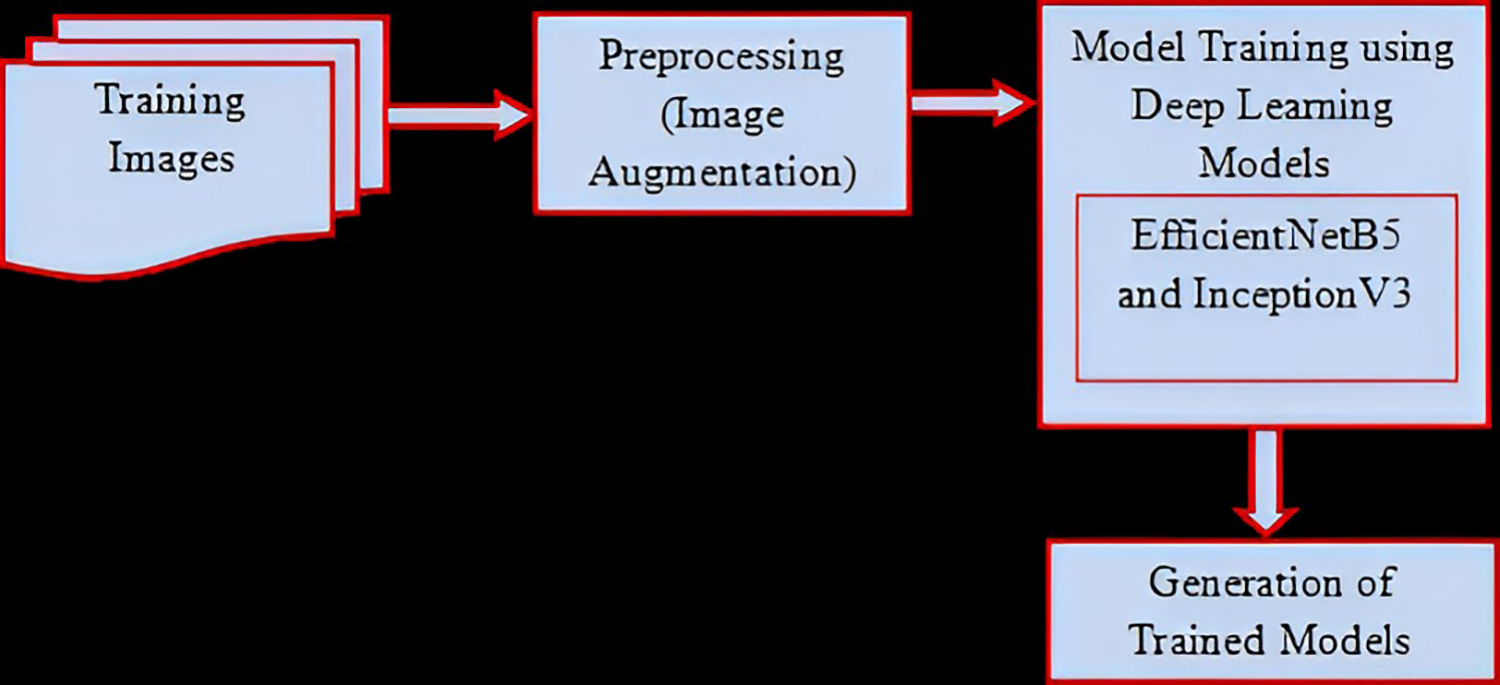
Training process.

Following preprocessing, the training phase commenced, where the images were subjected to augmentation techniques to introduce variability into the dataset, thus improving the model's ability to generalize to unseen data (Figure 1). Hyperparameter optimization was then conducted to fine-tune the model parameters, enhancing its performance. During training, the images were fed into two deep learning architectures, namely EfficientNetB5 and InceptionV3, which served as feature extractors. These models extract meaningful features from the images, capturing the distinctive characteristics of elongated styloid processes.

**Figure 2:**
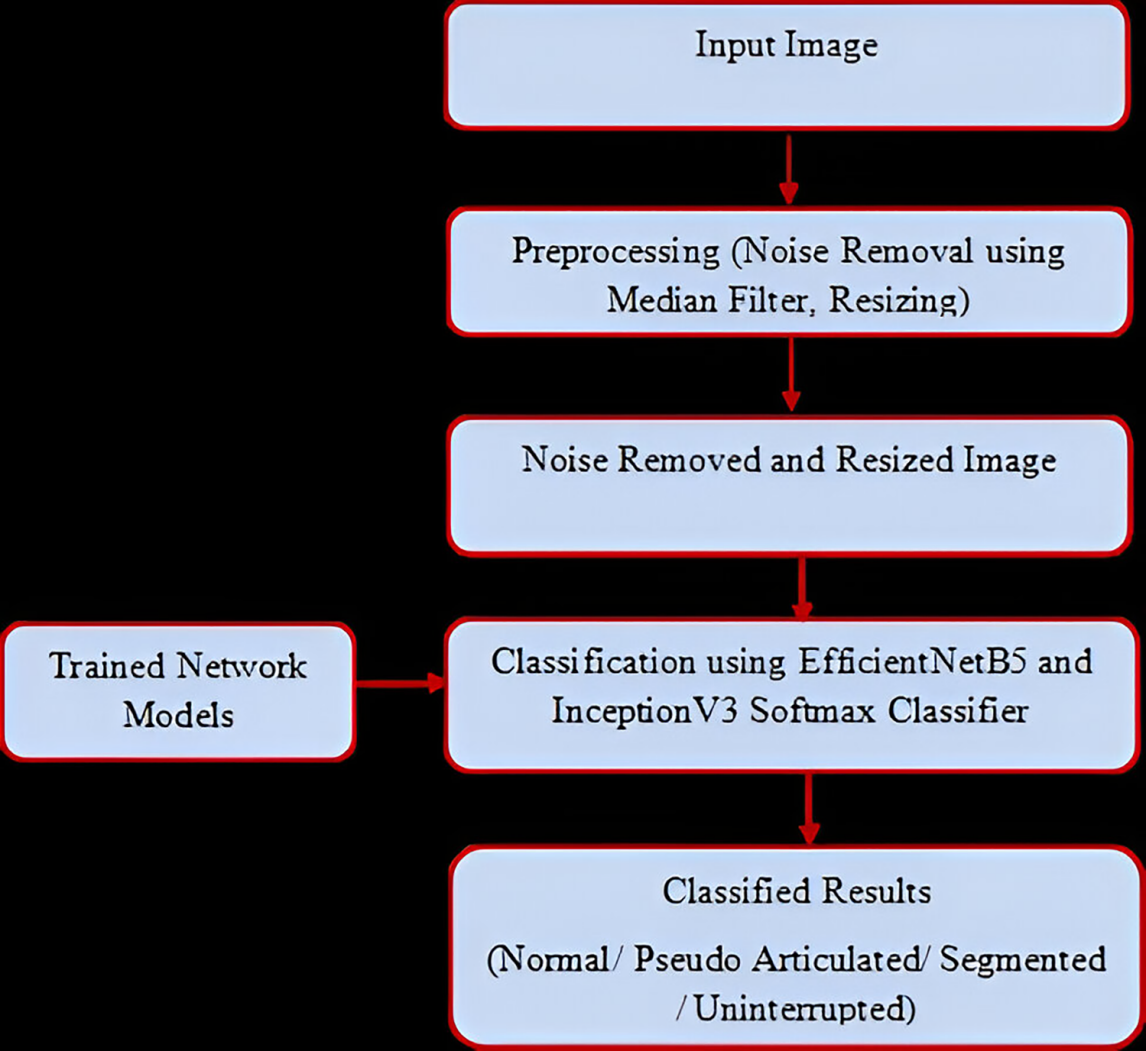
Testing process.

Once the models were trained, the inference phase begins. New input images undergo the same preprocessing steps as during training to maintain consistency. These preprocessed images were then passed through the trained EfficientNetB5 and InceptionV3 models, leveraging their softmax classifiers to classify the images into different types of elongated styloid processes based on the learned features (Figure 2).

Finally, the classification results obtained from both models were rigorously evaluated in terms of various performance metrics, including accuracy, precision, recall, and F1 score. Comparative analyses were conducted to assess the effectiveness of each model in accurately classifying elongated styloid processes. Overall, this methodology aimed to develop a robust system that can assist clinicians in diagnosing and planning treatment for patients with elongated styloid processes, thereby improving patient care outcomes.

## 3 Module implementation and description

The implementation of the present study was performed in step by step modules. The modules consisted of (1) Data Collection (2) Pre-processing (3) Training Phase (4) Inference Phase (5) Evaluation and Analysis. The system comprised of hardware and software requirements. The hardware comprised of System: Core i5 Processor, Hard Disk: 500 GB, Monitor: 15″ LED, Input Devices-Keyboard, Mouse, Ram—4 GB. The software included Operating system: Windows 10 and Coding Language: PYTHON (Jupyter Notebook).

### 3.1 Data collection

Real-time digital panoramic radiographs were collected from clinical sources to serve as the foundation for training and testing our deep learning models. These images capture the anatomical variations of elongated styloid processes and provide essential data for model development. By sourcing images from clinical settings, we ensured the relevance and applicability of our model to real-world scenarios. A comprehensive approach was followed to categorize different forms of elongated styloid processes. This involved meticulously gathering and scrutinizing 938 orthopantomograms (OPGs) sourced from clinical data. Using advanced ImageJ software from the National Institute of Health in Maryland, USA, we precisely measured the length of the styloid process in each case. Subsequently, we curated a dataset consisting of 330 images both unilaterally and bilaterally showcasing elongated styloid processes and 120 images displaying normal styloid processes. A carefully selected dataset of images was categorized based on Langlais et al.'s classification into “Normal,” “Pseudo Articulated,” “Segmented,” or “Uninterrupted.” These images were then separated into groups representing bilateral elongated styloid processes, left-side elongation only, and right-side elongation only. In the process of image preparation, the single image showing bilateral elongation of the styloid process was treated as two separate images after cropping.

### 3.2 Preprocessing

Before model training began, a critical preprocessing step was undertaken to enhance the quality of the images and remove any irrelevant details. This process involved applying a median filter for noise removal, which helped to clean up the images and improve their clarity. Additionally, the images were resized to a standardized dimension (Input Image Size − 128 × 128) to ensure consistency across the dataset. This preprocessing step was considered crucial for preparing the images for subsequent analysis and ensures that the deep learning models receive clean and uniform input data.

### 3.3 Training phase

The training phase began after preprocessing, where the images underwent augmentation techniques to introduce variability into the dataset. This augmentation process helped to enhance the model's ability to generalize to unseen data and improve its robustness, fine-tune the model parameters such as batch sizes (32), leveraging the ADAM optimizer, and epochs (50), ensuring optimal performance. During training, the images were fed into two deep learning architectures, EfficientNetB5 and InceptionV3, which served as feature extractors. These models extract meaningful features from the images, capturing the distinctive characteristics of elongated styloid processes.

### 3.4 Inference phase

Once the models were trained, the inference phase began. New input images underwent the same preprocessing steps as during training to maintain consistency. These preprocessed images were then passed through the trained EfficientNetB5 and InceptionV3 models. Leveraging their softmax classifiers, the models classified the images into different types of elongated styloid processes based on the learned features. This phase allowed for the application of the trained models to real-world images, enabling the automated classification of elongated styloid processes.

## 4 Proposed algorithm

### 4.1 EfficientNetb5

**Figure 3:**
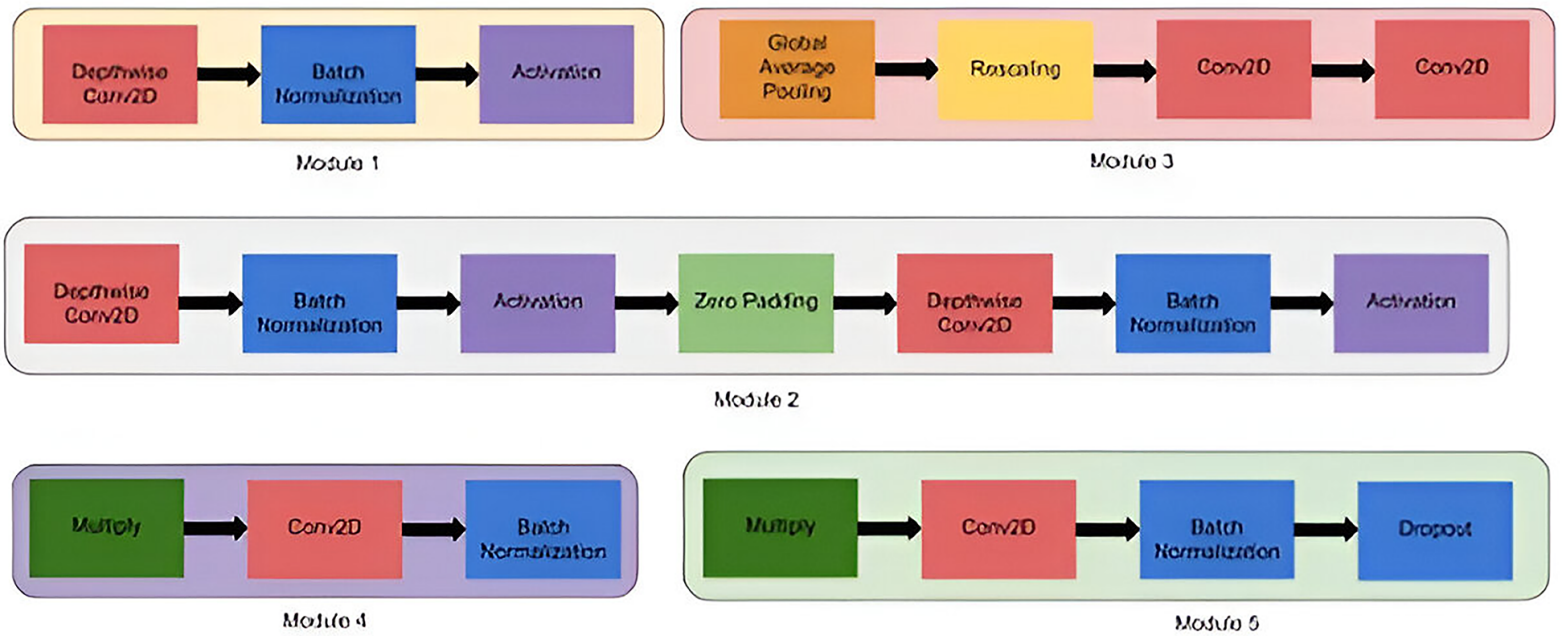
Architectural structure of EfficientNetB5.

EfficientNet is a convolutional neural network architecture and scaling technique that uniformly adjusts depth, width, and resolution using a compound coefficient. Unlike traditional methods that independently scale these dimensions, EfficientNet maintains uniform scaling by utilizing predefined coefficients. This approach ensures a principled and consistent scaling of network width, depth, and resolution across different models. The compound scaling method is justified by the intuition that if the input image is bigger, then the network needs more layers to increase the receptive field and more channels to capture more fine-grained patterns on the bigger image (Figure 3).

### 4.2 Inception V3

**Figure 4:**
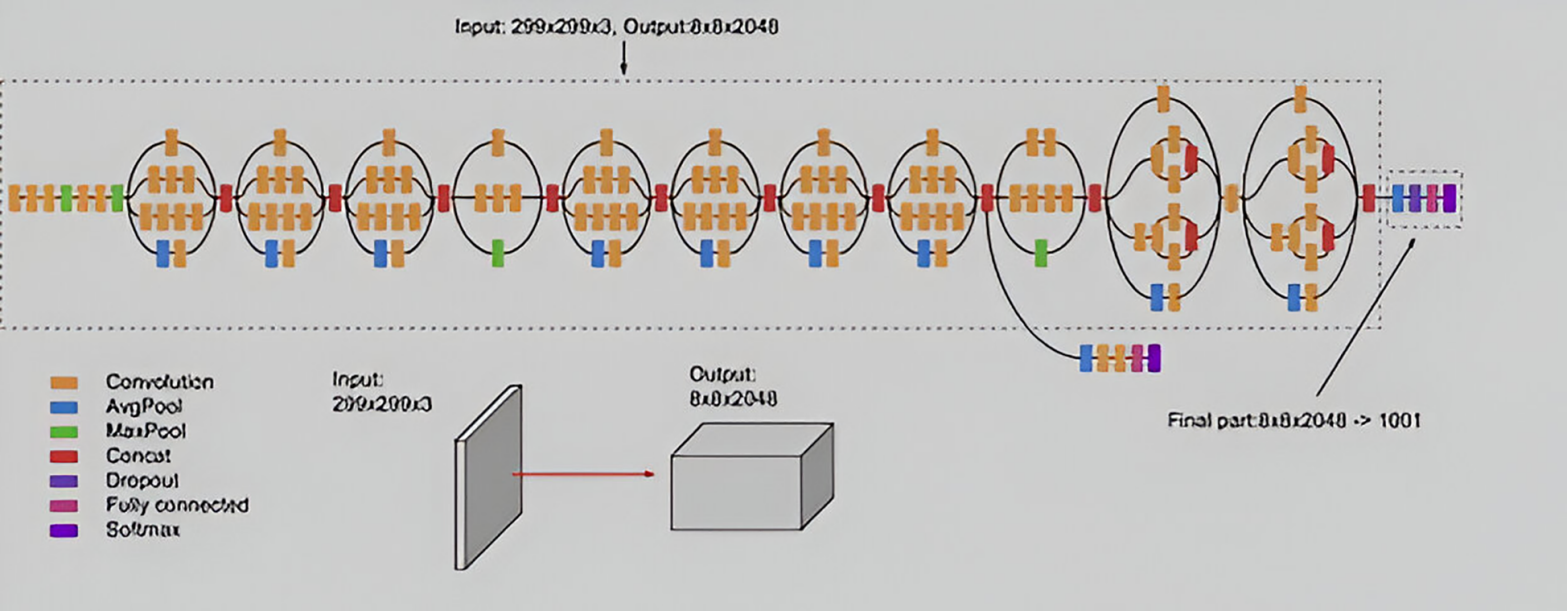
Architectural structure of Inception V3.

The Inception V3 is a convolutional neural network model designed for image classification, representing an advanced version of the original Inception V1 model from 2014. Developed by Google, Inception V3 incorporates several optimizations and techniques to enhance model performance. It features improved efficiency and computational cost-effectiveness compared to its predecessors. Despite its deeper architecture, Inception V3 maintains speed and includes auxiliary classifiers for regularization purposes. Released in 2015, Inception V3 comprises 42 layers and achieves lower error rates. Key enhancements in Inception V3 include factorization into smaller convolutions, spatial factorization using asymmetric convolutions, utilization of auxiliary classifiers, and efficient grid size reduction (Figure 4).

## 5 Results

### 5.1 Evaluation and analysis

• The classification results obtained from both models were rigorously evaluated in terms of various performance metrics, including accuracy, precision, recall, and F1 score by comparing the predicted labels with the ground truth labels.
• Accuracy: It measures the analysis of TP and TN to the total no. of test images.
  $$\mathrm{Accuracy}=\frac{TP+TN}{TP+TN+FP+FN}$$
• Precision: It is the estimation analysis of true positive to the aggregate value of true positive and false positive rate. It is given in eqn.
  $$\mathrm{Precision}=\frac{\left(TP\right)}{\left(TP+FP\right)}$$
• Recall: It is the estimation analysis of true positive rate to the aggregate value of the true positive and false negative rate. It is given in eqn.
  $$\mathrm{Recall}=\frac{\left(FP\right)}{\left(TP+FN\right)}$$
• F1-Score: F-Measure is the harmonic mean of recall and precision. Precision and recall are given equal weight in the standard F-measure (F1).
  $$F_{s}(1+\varepsilon^{2})\frac{\mathrm{Recall}\times \mathrm{Precision}}{\varepsilon^{2}\cdot \mathrm{Precision}+\mathrm{Recall}}$$

### 5.2 Training process

**Figure 5:**
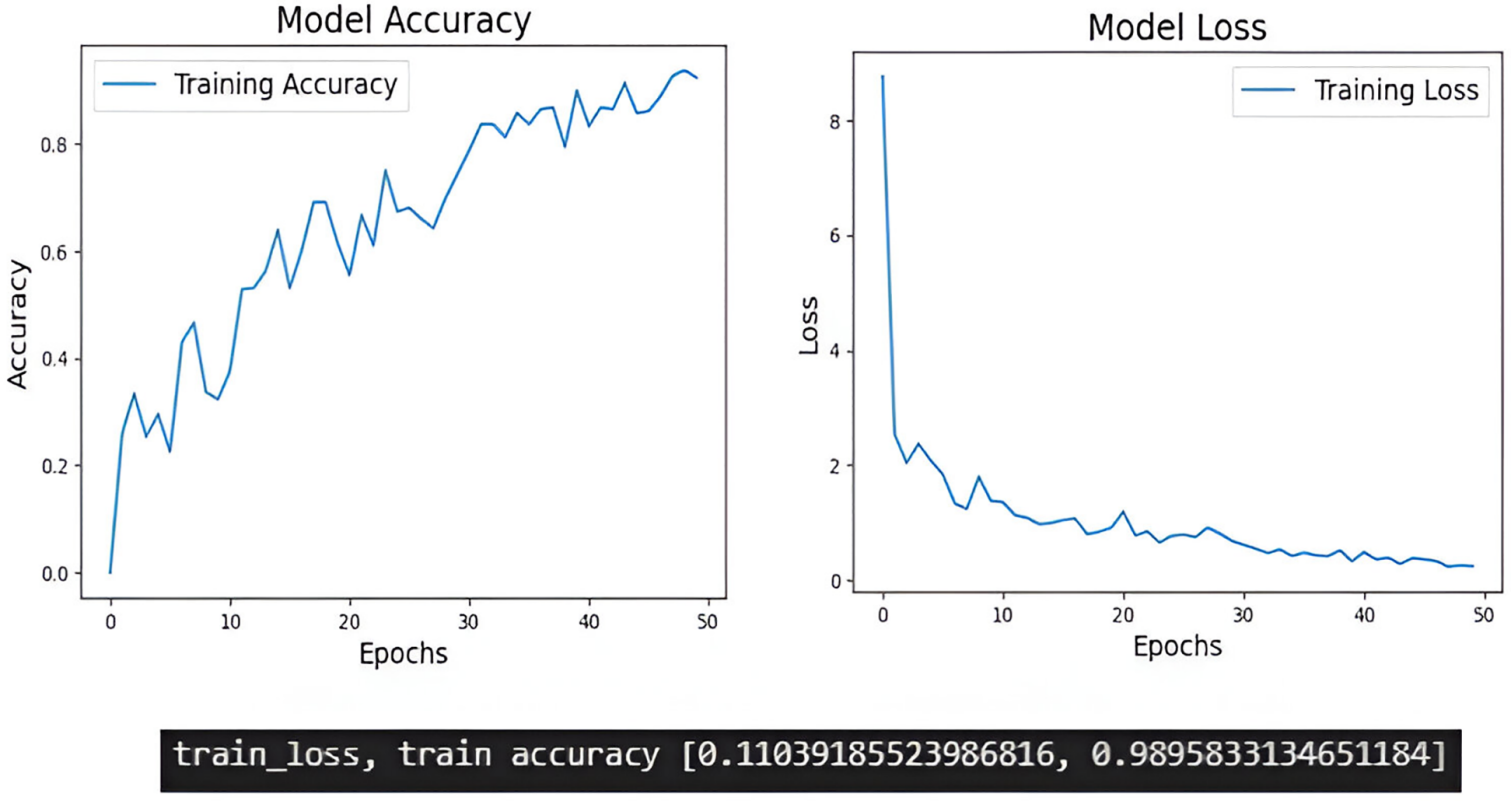
The training accuracy and training loss of Efficient NetB5.

**Figure 6:**
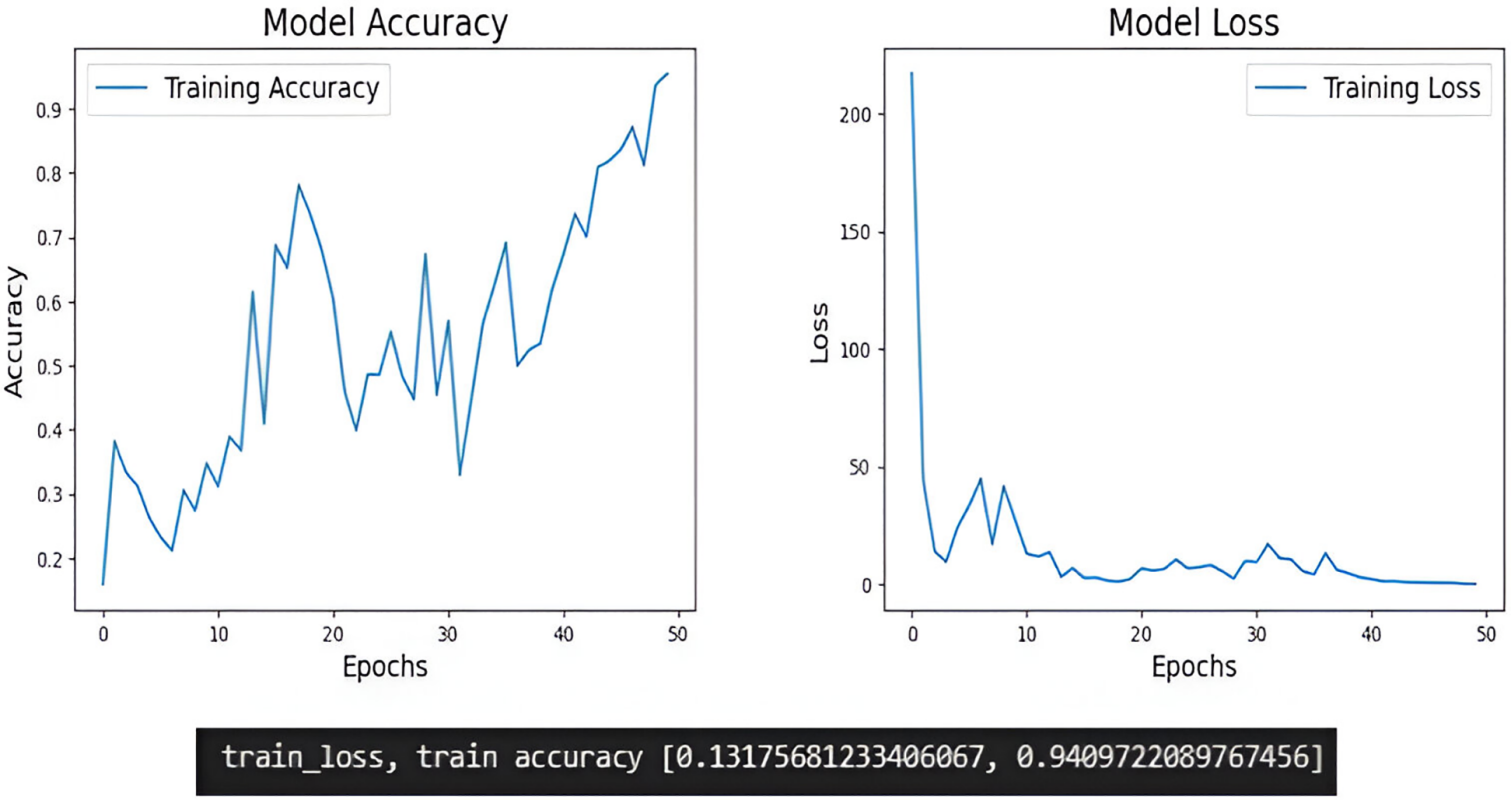
The training accuracy and training loss of Inception V3.

The training process of deep learning models, monitor the evolution of training accuracy and loss and is essential for assessing model performance and diagnosing potential issues such as overfitting or underfitting. Compared to Inception V3 proper training of the models were obtained in EfficientNetB5 (Figures 5, 6).

## 6 Testing process

**Figure 7:**
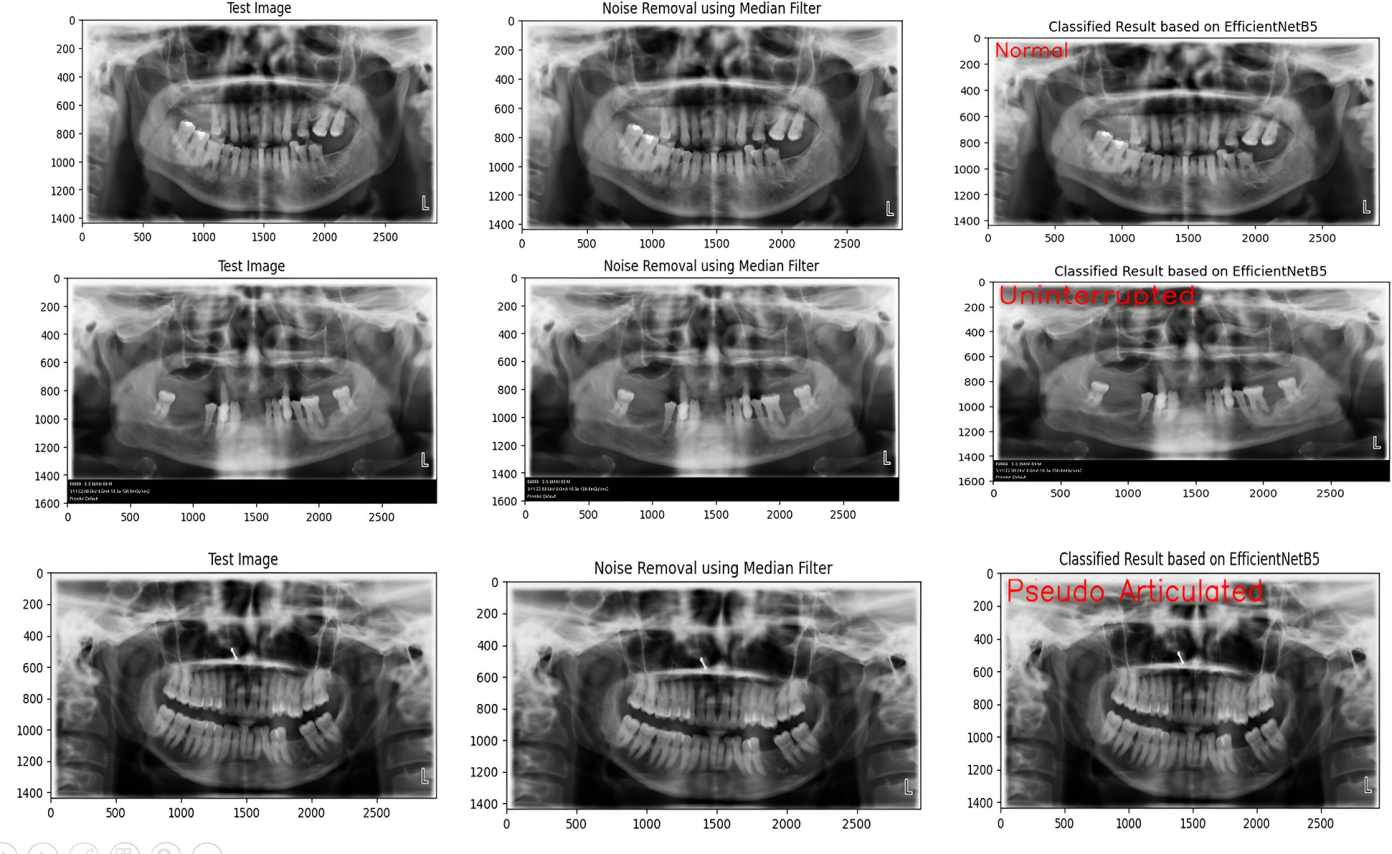
Efficient NetB5 model's correct identification of the lesion according to the classification.

These preprocessed images were passed through the trained EfficientNetB5 and InceptionV3 models, and the images into different types of elongated styloid processes based on the learned features were obtained (Figure 7).

## 7 Performance measure

### 7.1 Confusion matrix

**Figure 8:**
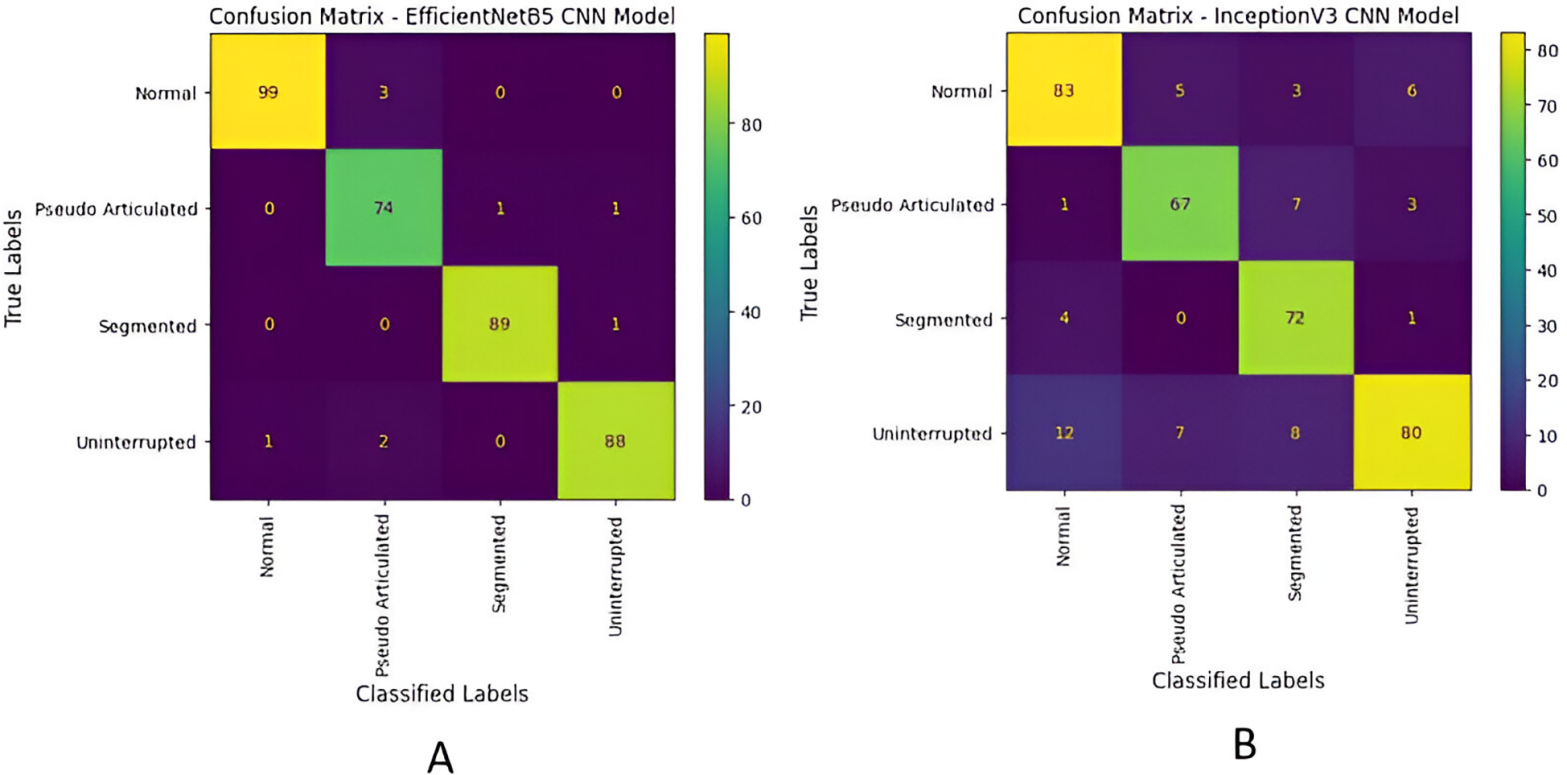
Confusion matrix of each model in predicting: “Normal”, “Pseudo Articulated”, “Segmented”, and “Uninterrupted” using the photographs in the test dataset. **(A)** EfficientNetB5, **(B)** Inception V3.

The confusion matrix was obtained which shows the classification performance of an EfficientNetB5 and Inception V3 models trained to categorize elongated styloid processes into four distinct classes: “Normal”, “Pseudo Articulated”, “Segmented”, and “Uninterrupted”. Each cell in the matrix represents the count of instances predicted by the model for a given combination of true and predicted classes (Figure 8).

### 7.2 Performance metrics—accuracy, precision, recall and F1-score

**Figure 9:**
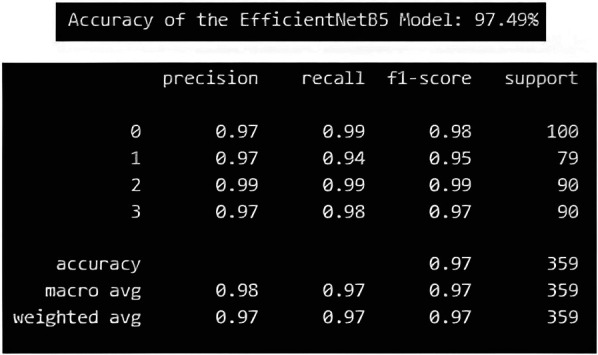
Performance metric for EfficientNetB5 model.

**Figure 10:**
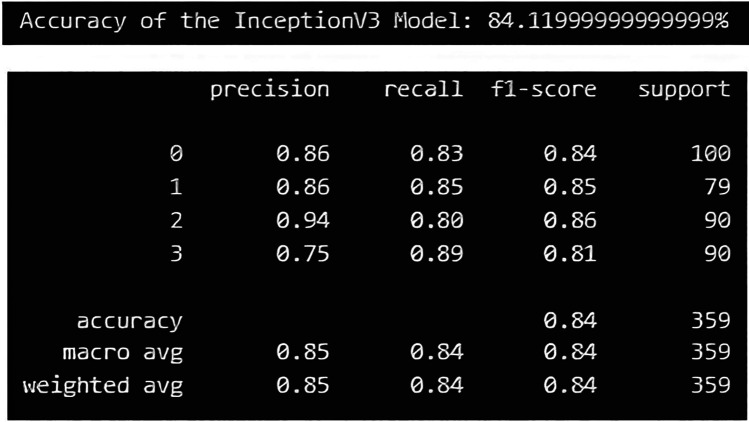
Performance metric for InceptionV3 model.

The EfficientNetB5 model achieved an accuracy of 97.49%, and a precision of 98.00%. Additionally, the model exhibited a recall of 97.00%, and a F1-score, of 97.00%, representing model's overall performance (Figure 9). The InceptionV3 model achieved an accuracy of 84.11%, and precision of 85.00%.Additionally, the model exhibited a recall of 84.00%, and F1-score of 84.00% (Figure 10).

**Figure 11:**
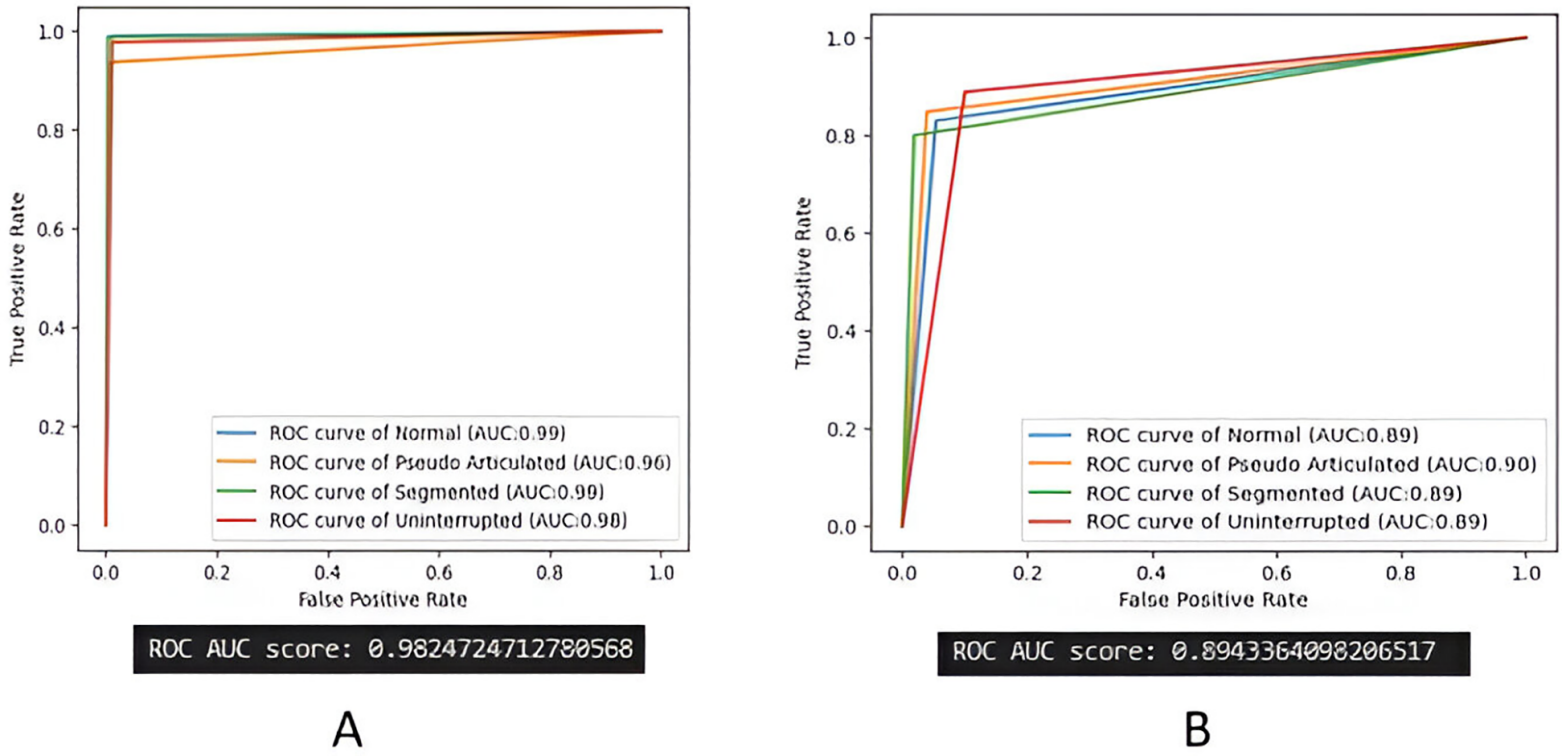
ROC curve and AUC analysis. **(A)** EfficientNetB5, **(B)** Inception V3 models.

Figure 11 shows the ROC curve and AUC values for both EfficientNetB5 and Inception V3 models. The AUC value of 0.9825 was obtained in EfficientNetB5 and AUC value of 0.8943 was obtained in Inception V3 models.

## 8 Discussion

The styloid process of the temporal bone serves as a point for several attachments, including the stylopharyngeus, stylohyoid, and styloglossus muscles, as well as the stylomandibular and stylohyoid ligaments. The elongated styloid process (ESP) is a common anatomical variation characterized by the elongation of the styloid process of the temporal bone sometimes causing Eagle's syndrome, leading to symptoms like dull pharyngeal pain, earache, a sensation of a foreign body in the throat, and difficulty in swallowing. Occasionally, a palpable mass in the tonsillar fossa exacerbates symptoms. Diagnosing this condition is challenging because of its proximity to other structures, requiring detailed history, clinical assessment, and imaging to differentiate it from conditions like neuralgias and temporomandibular disorders (15). Accurate classification of elongated styloid processes into different types based on their morphology is essential for appropriate clinical management, including diagnosis and treatment planning. Recently, there has been a surge in using advanced computational methods, especially deep learning like CNNs, to automate medical image classification (16). AI technology offers significant improvements in diagnosing elongated styloid processes by providing accurate, consistent measurements of styloid length and shape. This precision reduces the subjectivity and potential errors of manual assessments, especially useful in settings with limited health care work force. AI also aids in detecting subtle abnormalities, supporting early diagnosis of related degenerative changes. These models excel at extracting key features from images, leading to accurate classification of intricate patterns. Leveraging deep learning holds promise for creating faster and more dependable systems for classifying elongated styloid processes. Overall, AI-assisted evaluations enhance diagnostic accuracy, streamline clinical decision-making, and improve treatment planning, which is particularly valuable for surgical cases requiring a detailed understanding of anatomy.

Deep Learning, a branch of machine learning, concentrates on teaching artificial neural networks to execute intricate tasks akin to human brain information processing. Its prowess lies in automatically grasping patterns and features from data, empowering machines to forecast, decide, classify, and even anticipate treatment outcomes. This technology has garnered considerable acclaim and achievements across diverse fields with diagnosis being the most prevalent application. The present study was performed to develop a robust deep learning-based classification system capable of accurately categorizing different types of elongated styloid processes using digital panoramic radiographs. So the performance of two distinct deep learning architectures, namely EfficientNetB5 and InceptionV3 was used, in classifying elongated styloid processes, assessing their accuracy, precision, recall, and F1 score. Both these algorithms have been tried in diagnosis of various diseases in the medical field (17–19). To the best of our knowledge, this is the first study to explore AI's ability to be a reliable tool in detecting and classifying the elongation of styloid process.

During the training process these models discern unique features within images, capturing the defining traits of elongated styloid processes. These metrics are typically plotted over epochs, representing the number of passes through the entire training dataset. The training accuracy graph illustrates how well the model is learning to classify the training data correctly, whereas the training loss graph shows the evolution of the loss function, which quantifies the difference between the predicted and actual values. In the training accuracy graph, as epochs progress, we expect to see an increase in accuracy, indicating that the model is learning to make more accurate predictions on the training data. Conversely, in the training loss graph, we aim to observe a decreasing trend over epochs. A decreasing loss indicates that the model is minimizing the discrepancy between its predictions and the actual values, thus improving its performance. In our study EfficientNetB5 showed better training of the models compared to Inception V3.

The EfficientNetB5 and InceptionV3 model achieved an accuracy of 97.49% and 84.11% respectively, indicating the proportion of correctly classified instances across all classes. The precision of 98.00% in EfficientNetB5 and 85.00% in InceptionV3 highlights the model's ability to accurately identify positive instances within each class while minimizing false positives. Moreover, the models demonstrated a commendable recall rate of 97.00% in EfficientNet V5 and 84.00% in InceptionV3, underscoring its proficiency in identifying and capturing positive instances within the dataset. The F1-score, achieved at 97.00% for EfficientNetB5 and 84.00% for InceptionV3, is a metric that combines precision and recall into a single value. It offers a balanced assessment of the model's performance by considering both false positives and false negatives. These metrics collectively demonstrate the robustness and effectiveness of the classification model in accurately diagnosing different types of elongated styloid processes, thus showcasing its potential clinical utility in improving patient care outcomes. Similar studies using EfficientNetB5 and InceptionV3 models were tried earlier for classifying various diseases and both have shown promising results (17, 20).

The Receiver Operating Characteristic (ROC) curve and Area Under the Curve (AUC) analysis provide additional insights into the classification model's performance for distinguishing between the four classes of elongated styloid processes. The ROC curve visually represents the trade-off between the true positive rate (sensitivity) and false positive rate (1-specificity) across various thresholds. The AUC value of 0.9825 was obtained in EfficientNetB5 and AUC value of 0.8943 was obtained in Inception V3 models indicates the overall discriminative ability of the model across all classes, with higher values indicating better performance. This analysis underscores the model's effectiveness in accurately classifying elongated styloid processes and its potential clinical significance in aiding clinicians in diagnosing and managing patients with such conditions.

A recent similar study aimed to assess the precision of artificial intelligence in identifying elongation of the styloid process in digital panoramic radiographs. Additionally, it sought to compare the effectiveness of three distinct AI algorithms- logistic regression, neural network, and Naïve Bayes algorithms in Orange software against manual radiographic evaluation conducted by radiologists. A total of 400 digital panoramic radiographs (OPGs) were analyzed, and three different AI models were employed for styloid process elongation detection. Performance evaluation included accuracy, sensitivity, specificity, precision, recall, F1 score, and AUC- ROC. Results showed logistic regression and neural network algorithms achieving 100% accuracy without any false positives or false negatives, scoring 1.000 across all metrics. The Naïve Bayes model, while not as accurate, classified with an AUC score of 78%, performing better than random guessing despite 49 false positives and 59 false negatives. Thus the study concluded that logistic regression and neural network algorithms demonstrated accurate detection of styloid process elongation compared to manual radiographic evaluation. While the Naïve Bayes algorithm showed less accuracy, it still outperformed random guessing (21).

The present study presents a deep learning-based approach for automating the classification of elongated styloid processes using digital panoramic radiographs. Leveraging the advanced deep learning architectures EfficientNetB5 and InceptionV3, we have demonstrated their effectiveness in accurately categorizing elongated styloid processes into distinct types based on their morphological characteristics. Our experimental results reveal that the EfficientNetB5 model achieved an impressive accuracy of 97.49%, outperforming the InceptionV3 model, which attained an accuracy of 84.11%. This indicates the superior performance of EfficientNetB5 in accurately classifying elongated styloid processes. The high accuracy achieved by both models underscores the potential of deep learning techniques in enhancing the efficiency and accuracy of medical image analysis tasks. By automating the classification process, our proposed system offers several advantages over traditional manual methods, including reduced reliance on subjective interpretation and improved consistency in diagnosis. Our findings suggest that the integration of deep learning models, particularly EfficientNetB5, into clinical practice, could significantly improve diagnostic accuracy and streamline patient care processes.

## 9 Conclusion

Our study successfully developed and validated an automated system for detecting and classifying elongated styloid processes using deep learning models applied to digital orthopantomograms. This AI-based approach demonstrated high diagnostic accuracy, showing significant promise in accurately identifying and categorizing elongated styloid processes across radiographic images. Such technology represents a major step forward in automating diagnostic workflows, providing more reliable and efficient outcomes. Looking ahead, there is considerable potential for this technology to be implemented in clinical settings, where it could enhance diagnostic efficiency and consistency across patient cases, ultimately improving patient care.

## Data Availability

The raw data supporting the conclusions of this article will be made available by the authors, without undue reservation.
